# Resveratrol enhances the antimicrobial effect of polymyxin B on *Klebsiella pneumoniae* and *Escherichia coli* isolates with polymyxin B resistance

**DOI:** 10.1186/s12866-020-01995-1

**Published:** 2020-10-12

**Authors:** Li Liu, Jingyi Yu, Xiaofei Shen, Xingwei Cao, Qing Zhan, Yinjuan Guo, Fangyou Yu

**Affiliations:** 1 Department of Laboratory Medicine, The First Affiliated Hospital of Wenzhou Medical University, Wenzhou, 325000 China; 2 Department of Respiratory Medicine, The First Affiliated Hospital of Wenzhou Medical University, Wenzhou, 325000 China; 3 Jiangxi Provincial Key Laboratory of Medicine, Clinical Laboratory of the Second Affiliated Hospital of Nanchang University, Nanchang, 330006 China; 4 Jiangxi Provincial Key Laboratory of Preventive Medicine, Nanchang University, Nanchang, 330006 China; 5 Department of Clinical Laboratory Medicine, Shanghai Pulmonary Hospital, Tongji University School of Medicine, Shanghai, 200082 China; 6 Shanghai Key Laboratory of Tuberculosis, Shanghai Pulmonary Hospital, Tongji University School of Medicine, Shanghai, 200082 China

**Keywords:** Polymyxin B, Resveratrol, Combination therapy, Multidrug resistance (MDR)

## Abstract

**Background:**

Multidrug resistant (MDR) Gram-negative bacterial infections are a serious threat to human health due to the lack of effective treatments. In this study, we selected 50 Gram-negative bacterial strains, including 26 strains of *Klebsiella pneumoniae* and 24 strains of *Escherichia coli*, to explore whether resveratrol and polymyxin B have a synergistic killing effect.

**Results:**

MIC values against polymyxin B were ≥ 4 μg/mL for 44 of the strains and were 2 μg/mL for the other 6 strains. MICs against polymyxin B in the isolates tested were significantly reduced by the addition of resveratrol. The degree of decline depended on the bacteria, ranging from 1/2 MIC to 1/512 MIC, and the higher the concentration of resveratrol, the greater the decrease. Checkerboard analysis indicated a synergistic effect between resveratrol and polymyxin B; the optimal drug concentration for different bacteria was different, that of resveratrol ranging from 32 μg/mL to 128 μg/mL. Subsequent time-kill experiments showed that a combination of polymyxin B and resveratrol was more effective in killing bacteria.

**Conclusions:**

Our in vitro studies have shown that resveratrol can increase the sensitivity of MDR bacterial strains to polymyxin B, suggesting a potential new approach to the treatment of MDR infections.

## Background

In recent years, the emergence of Gram-negative bacteria that are resistant to multiple antibiotics has put a lot of pressure on healthcare centers around the world [1]. Infections caused by multidrug-resistant (MDR) Gram-negative bacteria not only have a higher mortality rate [2], but also impose greater economic burdens than infections caused by susceptible Gram-negative bacteria [3]. New antibiotics or more effective therapies are therefore urgently needed to solve this problem. In many situations, colistin and polymyxin B are considered the last antibiotics of choice [4].

Polymixin B has high affinity for the lipopolysaccharides (LPS) of Gram-negative bacilli and has been re-applied in the clinic. Polymixin B induces LPS aggregation, increasing the charge on cell membrane surfaces, and making it internalize and bind to the cell membrane, resulting in the leakage of cell contents [5]. Polymyxin B interacts directly with the lipid A component of lipopolysaccharide, thereby increasing the permeability of bacterial cell membranes [6]. Plasmids carrying anti-polymyxin B genes, including *mcr-1*, *mcr-2* and *mcr-3*, have been reported [7]; however, polymyxin combination therapy can improve bacterial killing and prevent the emergence of drug resistance [8].

Resveratrol is a natural polyphenolic compound that is found in large amounts in grapes, peanuts, and other plant sources, and in red wine [9]. Reports indicate that resveratrol can be used as a phytoalexin against fungal infections, and that it is a promising multi-target anticancer drug for the prevention and treatment of cancer [10–12]. Recent studies have demonstrated that it has potent antibacterial activity [13], and that it can significantly enhance the effect of aminoglycoside antibiotics (such as tobramycin, gentamicin, amikacin and netilmicin) on *Pseudomonas aeruginosa* biofilms [14]. Studies have shown that resveratrol can inactivate ATP synthase, thereby enhancing the sensitivity of *S.aureus* to polymyxin B [15].

In this study, we selected 50 strains of *K. pneumoniae* (*Klebsiella pneumoniae*) and *E. coli* (*Escherichia coli*) to study whether resveratrol and polymyxin B have synergistic effects.

## Results

### Bacterial isolates

We selected the strains for this study from a hospital in 2018, and screened out the experimental strains using drug sensitivity tests. We selected 50 multi-drug resistant strains (26 strains of *K. pneumoniae* and 24 strains of *E. coli*) for analysis (Table 1); six strains were sensitive to polymyxin B, and 44 strains were resistant. Resistance to polymyxin B was divided into two major categories, either due to an *mcr-1* gene carrying plasmid, or due to other reasons. Resistance to polymyxin B in *E. coli* was due to the *mcr-1* gene, while only one strain of resistant *K. pneumoniae* carried the *mcr-1* gene, the reasons for resistance in the other strains being unknown.

**Table 1:** Strain information and minimum inhibitory concentrations (MIC) of polymyxin B and resveratrol against bacterial isolates in this study

Isolate	Source	MIC					Polymyxin susceptibility and mechanism of resistance
		Res	PB	PB in the presence of 32 μg/ml Res	PB in the presence of 64 μg/ml Res	PB in the presence of 128 μg/ml Res	
*Klebsiella pneumoniae*							
1	human	512	16	8	4	2	mcr-1
2	human	512	> 512	> 512	128	2	Uncharacterized
3	human	512	8	2	2	2	Uncharacterized
4	human	> 2048	4	2	1	1	Uncharacterized
5	human	> 2048	4	2	1	1	Uncharacterized
6	human	512	> 512	> 512	256	8	Uncharacterized
7	human	1024	8	2	2	2	Uncharacterized
8	human	> 2048	> 512	256	16	1	Uncharacterized
9	human	2048	4	1	0.5	1	Uncharacterized
10	human	2048	4	1	1	1	Uncharacterized
11	human	512	4	2	2	1	Uncharacterized
12	human	> 2048	16	1	1	1	Uncharacterized
13	human	2048	4	2	0.5	1	Uncharacterized
14	human	2048	4	2	1	1	Uncharacterized
15	human	2048	16	4	2	1	Uncharacterized
16	human	2048	32	4	4	0.5	Uncharacterized
17	human	2048	64	16	4	1	Uncharacterized
18	human	512	4	1	0.5	0.5	Uncharacterized
19	human	> 2048	4	1	0.5	0.5	Uncharacterized
20	human	512	256	128	32	1	Uncharacterized
21	human	2048	16	2	2	0.5	Uncharacterized
22	human	> 2048	2	0.5	0.5	1	Susceptible
23	human	512	2	1	0.5	1	Susceptible
24	human	2048	2	2	0.5	0.5	Susceptible
25	human	> 2048	2	1	0.5	1	Susceptible
26	human	> 2048	2	1	1	1	Susceptible
*Escherichia coli*							
27	animal	> 2048	4	2	2	1	mcr-1
28	animal	> 2048	4	2	2	1	mcr-1
29	animal	2048	> 512	64	8	4	mcr-1
30	animal	> 2048	4	2	2	1	mcr-1
31	animal	> 2048	4	2	2	1	mcr-1
32	animal	> 2048	> 512	> 512	256	32	mcr-1
33	animal	> 2048	4	2	2	1	mcr-1
34	animal	> 2048	4	2	2	1	mcr-1
35	animal	2048	4	4	2	1	mcr-1
36	animal	> 2048	4	2	2	1	mcr-1
37	animal	> 2048	4	2	2	1	mcr-1
38	animal	> 2048	4	2	2	1	mcr-1
39	animal	> 2048	> 512	> 512	128	8	mcr-1
40	animal	2048	32	16	4	1	mcr-1
41	animal	> 2048	4	2	2	1	mcr-1
42	animal	> 2048	4	2	2	1	mcr-1
43	animal	> 2048	> 512	> 512	256	2	mcr-1
44	animal	> 2048	4	2	2	0.5	mcr-1
45	animal	2048	64	4	4	0.5	mcr-1
46	animal	> 2048	4	2	2	0.5	mcr-1
47	human	2048	4	1	0.5	0.5	mcr-1
48	human	> 2048	4	2	1	1	mcr-1
49	animal	2048	32	8	2	1	Uncharacterized
50	human	2048	2	2	1	1	Susceptible

*PB* Stands for polymyxin B and Res stands for resveratrol

### Resveratrol may be able to increase sensitivity to polymyxin B

Table 1 shows the MIC values of the 50 strains against polymyxin B and resveratrol. The MIC value against resveratrol in all strains was ≥512 μg/mL, and the MICs of polymyxin B it were reduced in all strains after the addition of resveratrol. In *K. pneumoniae*, after adding 32 μg/mL of resveratrol, in addition to the sensitive strains, strain 15 and strain 2 were highly resistant to polymyxin B, their MIC values for polymyxin B being decreased. The range of the drop was from 1/2 MIC to 1/8 MIC. When 64 or 128 μg/mL of resveratrol was added, the MIC values of polymyxin B against all strains decreased, and the degree of decline increased with increasing concentration of resveratrol. The situation observed in *E. coli* was very similar to that observed in *K. pneumoniae*; resveratrol seems to have a similar effect on different strains, regardless of the source of the strain and the cause of resistance to polymyxin B. This suggests that resveratrol may be able to increase the sensitivity of strains to polymyxin B (Table 1).

### Polymyxin B and resveratrol have a synergistic effect

Chequerboard assays can be used to detect synergy between two drugs. The Fractional Inhibitory Concentration (FIC) in the 14 bacterial strains selected for this experiment was less than 0.5, indicating the presence of a synergistic effect between polymyxin B and resveratrol (Table 2).

**Table 2:** FIC index values for polymyxin B and resveratrol against MDR bacterial isolates

Isolate	FIC of Polymyxin B	FIC of Resveratrol	FIC index	Interpretation
1	0.25	0.125	0.375	Synergistic
3	0.25	0.125	0.375	Synergistic
5	0.25	0.125	0.375	Synergistic
7	0.25	0.03125	0.28125	Synergistic
10	0.25	0.0625	0.3125	Synergistic
11	0.25	0.125	0.375	Synergistic
12	0.125	< 0.0625	< 0.1875	Synergistic
15	0.125	0.03125	0.15625	Synergistic
18	0.25	0.0625	0.3125	Synergistic
19	0.25	0.0625	0.3125	Synergistic
22	0.25	0.0625	0.3125	Synergistic
23	0.25	0.25	0.5	Synergistic
47	0.125	0.0625	0.1875	Synergistic
50	0.25	0.03125	0.28125	Synergistic

### Time-killresults of polymyxin B and resveratrol against *K. pneumoniae* and *E. coli*

**Fig. 1:**
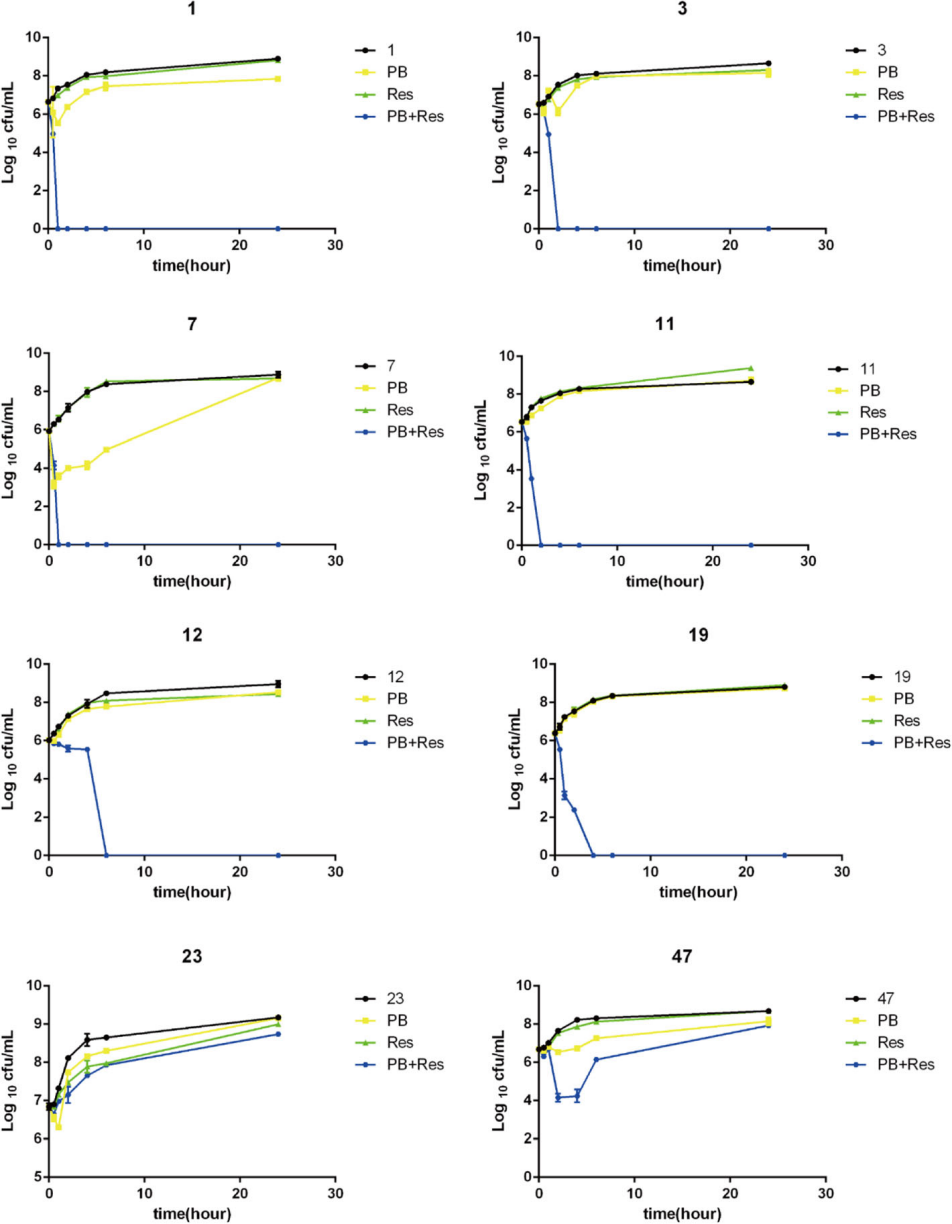
Time-kill experiments. Bacterial colony forming units in the absence of drug, and in the presence of 1/4 MIC polymyxin B, in the presence of 64 μg/ml resveratrol and in the presence of both drugs, after different periods of incubation PB = polymyxin B, Res = resveratrol. Data presented are Log_10_ CFU/mL mean values from the results of two independent experiments. Error bars represent standard deviations. Results for all 8 strains tested are presented (strain numbers are given above each figure panel)

We deduced the optimal concentration of resveratrol combined with polymyxin B from the chequerboard assays. With the exception of strains 12 and 47, the optimal concentration of polymyxin B for the other 13 bacterial strains tested when the concentration of resveratrol ranged from 32 to 128 μg/mL was 1/4 MIC polymyxin B. At a concentration of 128 μg/mL resveratrol, however, the DMSO in the drug solution exceeded 0.1% and was toxic to cells. For this reason, we chose the 1/4 MIC polymyxin B and 64 μg/mL intermediate concentration of resveratrol for time-kill experiments (Fig. 1). Some differences were observed between strains. 64 μg/mL resveratrol alone had no killing effect on bacteria. Strains 1 and 7 were killed after 1 h of treatment with the two drugs (1/4 MIC polymyxin B and 64 μg/mL resveratrol), strains 3 and 11 after 2 h, and strains 19 and 12 after 4 and 6 h respectively. Neither of the drugs alone had a significant killing effect on the strains tested. While strains 23 and 47 were not completely killed, the killing effect of the two drugs together was more pronounced in the early time than either drug alone. The number of colonies of these two strains reached the level of the untreated group at 24 h.

## Discussion

In view of the rapid emergence of multi-drug resistance and the general lack of new effective antibiotics developed in the last two decades, new methods for the treatment of MDR Gram-negative bacterial infections are urgently needed [1, 16]. Polymyxin B is considered to be the last choice of drug for the treatment of multi-drug resistant infections, but bacterial strains resistant to polymyxin B are increasing in prevalence [17]. Many studies have shown that other antibiotics or non-antibiotics combined with polymyxin B can improve its antibacterial activity [8, 18]. Resveratrol (trans-3,4′,5-trihydroxystilbene) is a polyphenolic compound that was first mentioned in an article in 1940 and isolated from the plant cucurbits by root separation [19]. Many reports indicate that resveratrol has an antiviral effect on HIV-1 [20] and herpes simplex virus [21, 22]. Resveratrol also has antibacterial effects [23]. In this study we explored the difference between resveratrol and polymyxin B combination therapy and monotherapy.

Resistance to polymyxin B in Gram-negative bacilli arises through different mechanisms, including alterations in their lipopolysaccharides, which have an overall negative charge and are the initial targets of polymyxin [24], activation of the PhoP/PhoQ and PmrA/PmrB two-component systems (TCS) by environmental stimuli, and specific mutations within the TCS also leads to constitutive activation and subsequent overexpression of the LPS modified gene [25–27]. In addition, the mutation/inactivation of the *mgrB* gene leads to the emergence of *K. pneumoniae* resistance [28]. The role of efflux in polymyxin resistance is unclear, but some studies have shown that efflux pumps are involved [29, 30]. In recent years, studies have shown that a plasmid-carried *mcr-1* gene is also involved in polymyxin resistance. The *mcr-1* gene encodes lipid A phosphoethanolamine transferase, an enzyme that inactivates polymyxin [31]. In general, polymyxin B drug resistance mechanisms are divided into two major classes: plasmid *mcr-1* mediated and non-plasmid mediated.

In this study, we found that combination therapy with polymyxin B and resveratrol is much more effective than monotherapy of either drug. MIC values are an indicator of drug activity. Our study found that the drug concentrations of polymyxin B and resveratrol required for combination therapy were much lower than those required for monotherapy. The combination of the two drugs had a similar effect on polymyxin B sensitive and resistant strains of *K. pneumoniae* and *E. coli*, suggesting that combination therapy with resveratrol and polymyxin B may have a relatively universal applicability. Checkerboard assays can be used to determine whether there is synergy between drugs. The FIC index of resveratrol and polymyxin B in the 14 strains selected for checkerboard analysis in our study (Table 2) was less than or equal to 0.5, indicating that resveratrol and polymyxin B have a synergistic antibacterial effect. The purpose of combination therapy is to increase the ability of the drugs to kill the bacteria, achieving a synergistic effect that the two antibiotics do not have when used alone [32]. We performed a time-kill experiment on 8 bacterial strains, finding that 6 of the strains were completely killed after 24 h of combined use of the two drugs. The growth of strains treated with monotherapies of the two drugs was almost the same as that of the untreated group. Although regrowth occurred in two strains resistant to polymyxin B, the combination of the two drugs still enhanced the initial bacterial killing and may thus help to remove bacteria from the body [8]. Our findings together indicate that the combination of polymyxin B and resveratrol significantly enhances bacterial killing. This is similar to the results of previous studies that resveratrol can enhance the sensitivity of S.aureus to polymyxin B [15].We speculate that resveratrol may destroy the cell envelope, allowing polymyxin B to bind to more targets in the bacterial outer membrane.

## Conclusions

In summary, our study has revealed a synergistic effect between resveratrol and polymyxin B. Resveratrol can increase the sensitivity of multi-drug resistant *K. pneumoniae* and *E. coli* to polymyxin B, enhancing the killing power of polymyxin B. We have only drawn a preliminary conclusion. Further experiments will be needed to substantiate our conclusion. Our findings may provide a potential method for the clinical treatment of multi-drug resistant Gram-negative bacilli infections. Further investigations of why resveratrol has different effects on polymyxin B sensitive and resistant strains and on the different causes of polymyxin B resistance are warranted.

## Materials and methods

### Bacteria strains and reagents

The strains used in this study were isolated from clinical samples and from animals, and included 26 strains of *K. pneumoniae* and 24 strains of *E. coli*. These strains were isolated from the First Affiliated Hospital of Wenzhou Medical University. Polymyxin B and resveratrol powder were purchased from Solarbio (Beijing, China). Polymyxin B and resveratrol were dissolved in deionized water and dimethyl sulfoxide (DMSO) to prepare stock solutions with a final concentration of 10 mg/mL and 100 mg/mL, respectively, and sterilized using a 0.20-μm cellulose acetate syringe filter. The stock solution was stored at − 20 °C for no more than 1 month.

### MIC assays

MIC determinations were performed by the broth microdilution method according to the Clinical and Laboratory Standards Association (CLSI) protocol [33]. MICs were determined in 96-well microtiter plates using freshly prepared Mueller-Hinton broth (Solarbio, Beijing, China). The final volume of bacterial samples was 200 μl, and the bacterial concentration was 5 × 10^^5^ CFU/ml. Microtiter plates were read visually after incubation at 37 °C for 20 h. *E. coli* ATCC 25922 was used as an internal quality control strain. As resveratrol has no CLSI breakpoint, and the CLSI breakpoint of *K. pneumoniae* and *E. coli* to polymyxin B has not yet been established by CLSI, therefore, the European Antimicrobial Susceptibility Testing Committee (EUCAST) showed that the breakpoint of Enterobacter to polymyxin B is 2 μg/mL (European Antimicrobial Susceptibility Testing Committee [EUCAST], 2020).

### Chequerboard assays

96-well sterile microplates were used for the checkerboard dilution assays. Each antibacterial drug was diluted with bactericidal MH broth to a maximum concentration of 2-times the MIC concentration of the drug. Eight concentrations of each drug were prepared by dilution. Fifty microliters of the appropriate drug dilution was added to the wells of the plates, together with 100 μl of the bacterial solution (giving a final inoculum of 5 × 10^5^ CFU/mL). MICs were recorded as the minimum drug concentration without bacterial growth. The interaction between the drugs was judged by calculating the FIC index according to the formula: FIC index = FIC_(drug A)_ + FIC_(drug B)_, where FIC = the MIC of the drug when in the combination/MIC of drug tested individually. FIC index values were interpreted as follows: “synergistic effects” = FIC index ≤0.5, “antagonism” = FIC index > 4.0, and “no interaction” = FIC index > 0.5–4.0 [34].

### Time-kill assays

Time killing experiments were performed using a slightly modified method [8]. Briefly, bacteria were grown overnight in 20 mL MHB (Mueller-Hinton Broth). The overnight broth culture was transferred to 20 mL of fresh MHB at a dilution of ~ 50–100 fold and incubated for an additional 3–4 h to produce log phase cultures of about 0.55 McFarland units. Log phase cultures were transferred to borosilicate glass tubes (to minimize non-specific binding to the plastic resulting in drug loss), diluted approximately 100-fold, and then transferred to 5 mL of fresh MHB for treatment. Polymyxin B, resveratrol or both compounds were added to the tubes as appropriate so that the final concentration of polymyxin B reached 2 μg/ml, and the final concentration of resveratrol reached 64 μg/ml. Samples were removed aseptically at 0, 0.5, 1, 2, 4, 6 and 24 h, serially diluted with physiological saline, and 10 μl of the bacterial sample was dropped on a blood agar plate. Colonies were counted after incubation at 37 °C for 24 h. Combinations of polymyxin B and resveratrol were considered synergistic if the bacteria kill ≥2 log_10_ compared to the most effective monotherapy.

### Determination of *mcr-1* gene

The polymerase chain reaction was used to amplify the *mcr-1* gene from each bacterial strain and the product was sent for commercial sequencing analysis. Sequencing results were compared and analyzed (Tsingke, Beijing, China), and *mcr-1* positive and negative strains were identified. Primers used in the experiment were *mcr-1*-F (5′- ATCAGCCAAACCTATCCC-3′) and *mcr-1*-R (5′- TAGACACCGTTCTCACCC-3′).

## Data Availability

The datasets generated during the current study are available from the corresponding author upon reasonable request. Most of the data is included in this published article.

## Abbreviations

*K. pneumoniae*: *Klebsiella pneumoniae*
*E. coli*: *Escherichia coli*
MIC: Minimum inhibitory concentration
LPS: Lipopolysaccharide
DMSO: Dimethyl sulfoxide
CLSI: Clinical and Laboratory Standards Institute
EUCAST: European Antimicrobial Susceptibility Testing Committee
FIC: Fractional inhibitory concentration
MHB: Mueller-Hinton Broth
TSB: Tryptic Soy Broth
PBS: Phosphate buffer solution
OD: Optical density
CFU: Colony-Forming Units
